# Whole-genome sequence analysis and probiotic characteristics of *Lactococcus lactis* Subsp*. lactis* strain Lac3 isolated from traditional fermented buffalo milk (Dadih)

**DOI:** 10.1186/s43141-023-00503-y

**Published:** 2023-05-02

**Authors:** Nshimiyimana Sylvere, Apon Zaenal Mustopa, Sri Budiarti, Lita Meilina, Ai Hertati, Ira Handayani

**Affiliations:** 1 School of Biotechnology, IPB University, Bogor, Indonesia; 2 Research Center for Genetic Engineering, National Research and Innovation Agency (BRIN), Bogor, 16911 Indonesia; 3 Indonesia Research Center for Bioresources and Biotechnology, IPB University, Bogor, Indonesia; 4 Research Center for Applied Microbiology, National Research and Innovation Agency (BRIN), Bogor, 16911 Indonesia

**Keywords:** Genome-sequencing, In vitro characteristics, Pathogenicity, Potential probiotic, *Lactococcus lactis*

## Abstract

**Background:**

Probiotics are live microorganisms that provide beneficial effects on the host’s health when exploited in adequate amounts. This study aimed at carrying out whole-genome sequence analysis and in vitro potential probiotic characteristics of *Lactococcus lactis* subsp*. lactis* strain Lac3 isolated from the spontaneously fermented buffalo milk named Dadih.

**Results:**

The results from de novo assembly indicated that the assembled genome consisted of 55 contigs with a genome size of 2,441,808 bp ~ (2.44 Mb), and GC % content of 34.85%. The evolution history result showed that the strain Lac3 was closely related to *Lactococcus lactis* species deposited in NCBI with a sequence similarity ≥ 99.93%. *L*. *lactis* subsp. *lactis* Lac3 was non-pathogenic with a probability of 0.21 out of 1 and had a pathogenicity score of zero (0), and neither harbored virulence factors nor acquired antibiotic resistance phenotypes. *L*. *lactis* subsp. *lactis* Lac3 exhibited the potential probiotic characteristics to tolerate acid at pH (2.0 and 5.0), salinity (1–5% NaCl), bile salt of (0.3–1.0%) and had auto-aggregation capacity increased from 6.0 to 13.1%.

**Conclusion:**

This study described a novel strain of *Lactococcus lactis subsp. lactis* called Lac3, which exhibits probiotic properties that could be beneficial in the development of probiotics.

## Background

Probiotics are living microorganisms that improve the health of the host system when consumed in sufficient amounts [1]. Health benefits provided by probiotics include the synthesis of vitamins, amino acids, short-chain fatty acids, stimulation of the immune system, production of bacteriocins, and metabolites with antioxidant activity, among others. Lactic acid bacteria (LAB) and Bifidobacteria are the best studied and widely applied as probiotic microorganisms in yogurt and various dairy products [2]. Several reasons contribute to the popularity of probiotics, including the fact that they are non-pathogens, harmless, and maintain adaptability in the gastrointestinal tract [3, 4]. On the other hand, other members of lactic acid bacteria, such as *Lactococcus lactis*, including *L. lactis* subsp. *cremoris* and *L. lactis* subsp. *lactis* has been used as starter cultures in the production of various fermented dairy products, including cheese and yogurt [5], and have been included in the GRAS (generally recognized as safe) status by FDA (the United States Food and Drug Administration) [6].

The potential of a probiotic to adapt to stress conditions, including acid in the stomach, bile salts in small intestines, and the production of antimicrobial compounds are essential characteristics for the probiotics to confer health benefits to the host. Furthermore, auto-aggregation characteristic indicates the potential of the probiotics to adhere to and colonize the gastrointestinal tract of the human [7].

According to the European Food Safety Authority Guidelines, selecting beneficial health and food-grade probiotics, probiotic candidates should be assessed concerning taxonomic identification, safety, and pathogenicity analysis or “adverse effects”, antibiotic resistance, and genome sequencing [8]. The technology of whole-genome sequencing (WGS) and genomic analysis have been the robust tools used to predict antimicrobial resistance genotypes in bacteria [9], stress tolerance, and adhesion mechanism [10].

In the study conducted by Mustopa et al. [11], *L. lactis* subsp*. lactis* Lac3 showed the ability to display antioxidant activity with an inhibitory capacity of 64.2%. However, there is no data regarding the whole-genome sequencing analysis, and the study regarding in vitro characteristics of the probiotic potential of *L*. *lactis* subsp*. lactis* Lac3 is less sufficient to select this strain Lac3 as a future probiotic candidate. For this reason, the current study aimed to advance understanding of the genome diversity, whole-genome sequencing analysis, and in vitro characteristics of probiotic potential of *L. lactis* subsp*. lactis* Lac3.

## Methods

### Identification of *L. lactis *subsp. *lactis* Lac3

*Lactococcus lactis* subsp*. lactis* strain Lac3 is one of the probiotic collections from the Research Center for Biotechnology, Indonesian Institute of Sciences (LIPI). The genotypic identification was conducted based on the 16S rRNA gene sequencing by performing the sequence comparison in which the consensus sequence generated was compared with other sequences available in the GenBank in the NCBI using the Basic Local Alignment Search Tool (BLAST). In addition, the cell morphology was identified using scanning electron microscopy (SEM). SEM analysis was conducted in accordance with previous reports with slight modification [12]. A single colony of *L. lactis* subsp. *lactis* Lac3 was grown in 5 mL of GM17 broth (Himedia, India) and subjected to an overnight incubation at 30 °C. Afterward, 1 mL cell culture was harvested by centrifugation at 6000 rpm for 10 min at 4 °C (Thermo Fisher Scientific Inc., USA). Subsequently, the pellets were prepared and then observed using a scanning electron microscope (JSM IT200, Japan).

### Genome sequencing, assembly, annotation, and bioinformatic analysis

The whole-genome sequencing was performed by (Novogene Co., Ltd., Japan) using the Illumina MiSeq Next-generation sequencer. We used the Quick-DNA™ HMW MagBead Kit to prepare the library before sequencing. The raw reads were subjected to the FastQC (version 0.11.8) [13]. Assembly quality assessment was checked using QUAST v5.0.2 integrated into the PathoSystems Resource Integration Center (PATRIC) platform [14]. The good-quality reads which we obtained from previous analysis using FastQC and QUAST were subjected to the de novo assembly using the Unicycler v. assembler algorithm integrated into the PATRIC. The annotation was added by Prokaryotic Genome Annotation Pipeline (PGAP) and Rapid Annotation using Subsystem Technology (RAST) [15]. The antibiotic resistance genes were predicted from the Comprehensive Antibiotic Resistance Database (CARD) and (ResFinder 4.1). Mobilome, including phages, was searched under PHASTER [16]. Mobile genetic elements (MGEs) and their relationships to the antimicrobial resistance genes and virulence factors, the insertion sequences (IS), plasmids, and the virulence-related factors were identified by the Center for Genomic Epidemiology (CGE) [16]. The Clustered Regularly Interspaced Short Palindromic Repeats (CRISPR)/Cas system was predicted using CRISPR/CasFinder [16].

### Phylogenetic tree analysis

The phylogenetic tree was constructed based on the 16S ribosomal RNA (16S rRNA) gene sequence. The obtained consensus sequence was compared with other sequences of closely related species retrieved from GenBank/NCBI and aligned using MUSCLE software. The evolutionary analyses were conducted in MEGA-X (version 10.2.6) [17]. The taxonomic connection was inferred using the Maximum Likelihood method and the Tamura-Nei model [18], and the bootstrap values were inferred from 1000 replications.

### In vitro assessment of probiotic characteristics of *L. lactis *subsp. *lactis* Lac3

#### Antibiotic susceptibility test (AST)

The antibiotic resistance was evaluated as described by Ramalho et al. [19] with modification. In brief, bacteria culture as much as 1.0% (v/v) were inoculated in 5 mL of de Man and Rogosa Sharpe broth (MRS) (Himedia, India) and incubated overnight at 37 ºC. After that, the bacteria cell culture was diluted ten-fold using 0.9% NaCl buffer saline (Merck, Darmstadt, Germany). The optical density between 0.08–0.1 equals 0.5 McFarland standard was measured at a wavelength of 600 nm. Subsequently, 100 μL of bacteria suspension was spread uniformly on solidified MRS agar (Himedia, India). Tetracycline (30 µg), ampicillin (10 µg), amoxicillin (30 µg), penicillin G (10 units), and chloramphenicol (30 µg) test discs (Thermo ScientificTM, OxoidTM Discs, Oxoid Ltd., UK) were laid aseptically on the surface of agar using sterilized forceps. After an anaerobic incubation at 37 ºC for 24 h, the inhibition-zone diameter around the discs was measured using a Vernier Caliper KENMASTER (150 × 0.02 mm). The results were interpreted as follows: resistant/R (≤ 15 mm); intermediate/I (16–20 mm); intermediate (I); sensitive/S (≥ 21 mm) [20]. This test was done in three replications, and the antibiotic-free blank discs were used as the negative control.

#### Tolerance to sodium chloride (NaCl)

Tolerance to NaCl was determined as previously described by Uymaz Tezel et al. [21] with modification. The GM17 broth (Himedia, India) was modified with different sodium chloride concentrations (0%, 1%, 2%, 4%, 5%, 6%) (w*/*v) was used. The fresh overnight culture was prepared by inoculating 1% (v/v) inoculum of *L. lactis subsp. lactis* Lac3 was inoculated into sterilized GM17 and incubated anaerobically overnight at 30 ºC. Subsequently, 2% (v/v) of the overnight culture was inoculated into fresh media and incubated at 30 ºC for 48 h. The absorbance was measured at 600 nm after 24 h and 48 h. The unmodified GM17 broth was considered a negative control, and the experiment was done in three replications.

#### Tolerance to bile salt

Susceptibility to bile salt was determined on GM17 agar as previously described by Yerlikaya et al. [22] with modification. In brief, as much as 1% (v/v) inoculum of *L. lactis* subsp*. lactis* Lac3 was inoculated into 5 mL of GM17 broth and incubated at 30 °C for 24 h. Afterward, 100 µL cells were resuspended in 900 µL 0.85% NaCl solution. Subsequently, 10 µL was dispensed on modified GM17 agar with different concentrations of bile salts (0%, 0.3%, 0.5%, 1%, and 2%) (w/v) using a glass spreader. The results were interpreted by observing the growth as “growth” or “no growth” after 96 h of incubation at 30 °C. This experiment was repeated three times, and the unmodified medium with bile salt was used as a negative control.

#### Acid tolerance

The *Lactococcus lactis* subsp*. lactis* Lac3 isolate was cultured on GM17 media at 30 °C overnight (16–18 h). The actively grown cells were harvested by centrifugation at 7000 rpm for 10 min at 4 °C (Thermo Fisher Scientific Inc., USA). The cells were inoculated in 5 mL of GM17 media with pH 2.5 and incubated at 30 °C, and GM17 media at pH 7.0 was considered as a control. After the time interval of 0, 1, and 2 h, samples were withdrawn and serially diluted using sterile 1 × phosphate-buffered saline (PBS). Viable cell colonies were enumerated by spreading 100 µL samples of the appropriate dilution on GM17 agar, and each sample was done in three replications. The viable colonies were counted by the Total Plate Count (TPC) method, and the results were expressed in log_10_ CFU/mL after 48 h [23]. Data were expressed as mean ± SD taken from three replications at (*p* < 0.05).

### Auto aggregation characteristics

As described by Yadav et al. [23], *L. lactis* subsp*. lactis* Lac3 was cultured for (16–18 h), and cells were harvested by centrifugation at 7000 rpm for 10 min at 4 °C. Afterward, the cells were washed three times using (1 × PBS). Furthermore, cells were suspended in (1 × PBS) and adjusted to an absorbance OD_600_nm ~ 0.5–0.6 using UV–visible spectrophotometer (Shimadzu, Japan). The cell suspension was incubated at 30 °C for 0, 2, 16 h, and at each time interval, 1 mL upper layer of the suspension was measured at 600 nm. The decrease in absorbance was expected as cell auto-aggregation and was expressed in percentage by the following formula:

$$\mathrm{\%\ Cells\ aggregation}=\frac{\mathrm{Initial\ OD}-\mathrm{Final\ OD}}{\mathrm{Final\ OD}}x 100$$

### Bioinformatics analysis

To predict a cluster gene that correlates to the probiotic properties of L. lactis subsp. lactis strain Lac3 using https://antismash.secondarymetabolites.org. In addition, we use ARTS (Antibiotic Resistant Target Seeker Version 2) (http://arts.ziemertlab.com/) to detect antibiotic resistance-encoding genes.

### Statistical analysis

All findings were interpreted as mean ± standard deviation (SD). The statistical package for social sciences (SPSS Inc., Chicago, USA) program version 22 was used to analyzed the data using the one-way analysis of variance (one-way ANOVA). Duncan's multiple range test (DMRT) was employed to determine the significance of the difference between means at a probability level of (*p* < 0.05).

## Results

### Genomic features and phylogenomic analysis

**Fig. 1:**
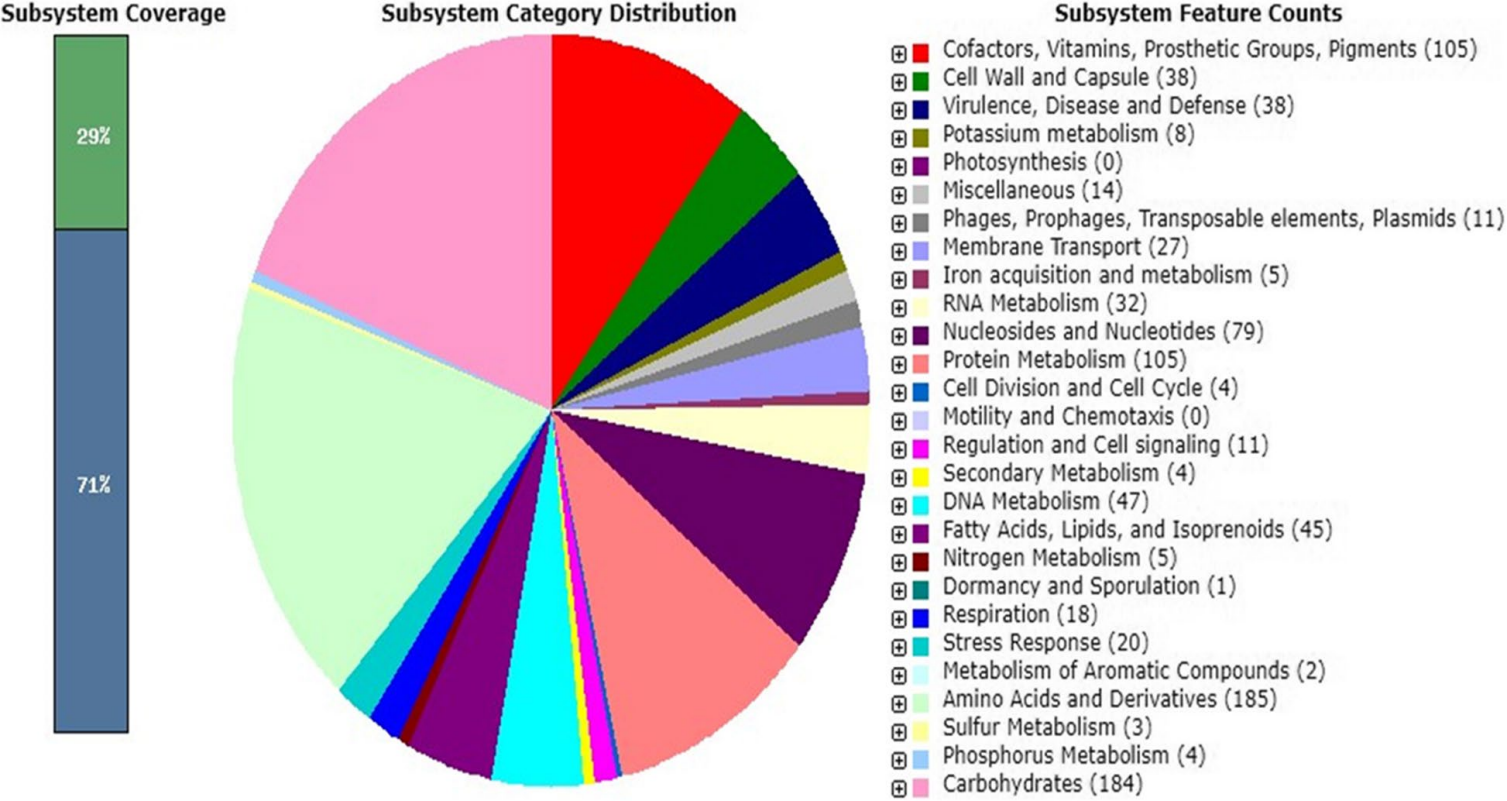
An overview of the subsystem categories annotated in the genome of *L. lactis* subsp*. lactis* Lac3 using RAST webserver

**Fig. 2:**
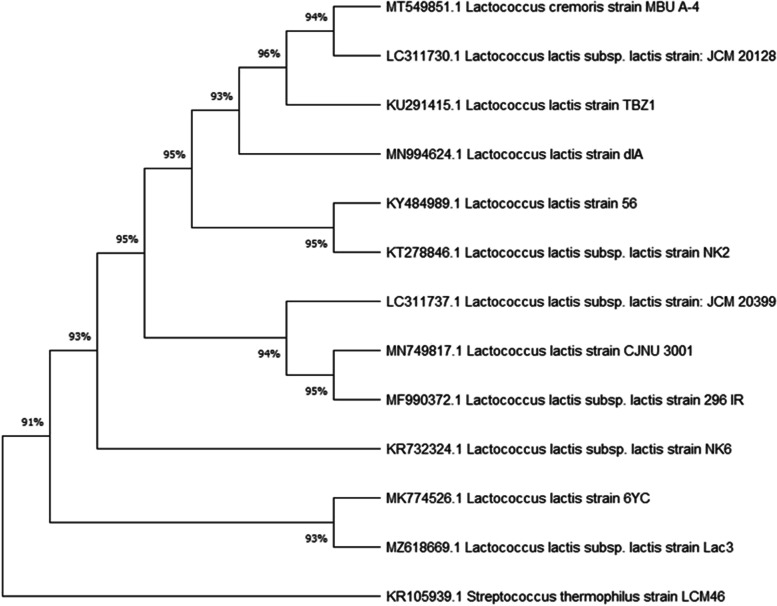
The phylogenetic tree was constructed based on 16S rRNA gene sequences showing a taxonomic connection of *L*. *lactis* subsp. *lactis* strain Lac3 (highlighted in yellow) with the closest hits retrieved from GenBank/NCBI. The evolutionary history was inferred by using the Maximum Likelihood method and the Tamura-Nei model. *Streptococcus thermophilus* strain LCM461 (KR105939) was added as an out-group

**Fig. 3:**
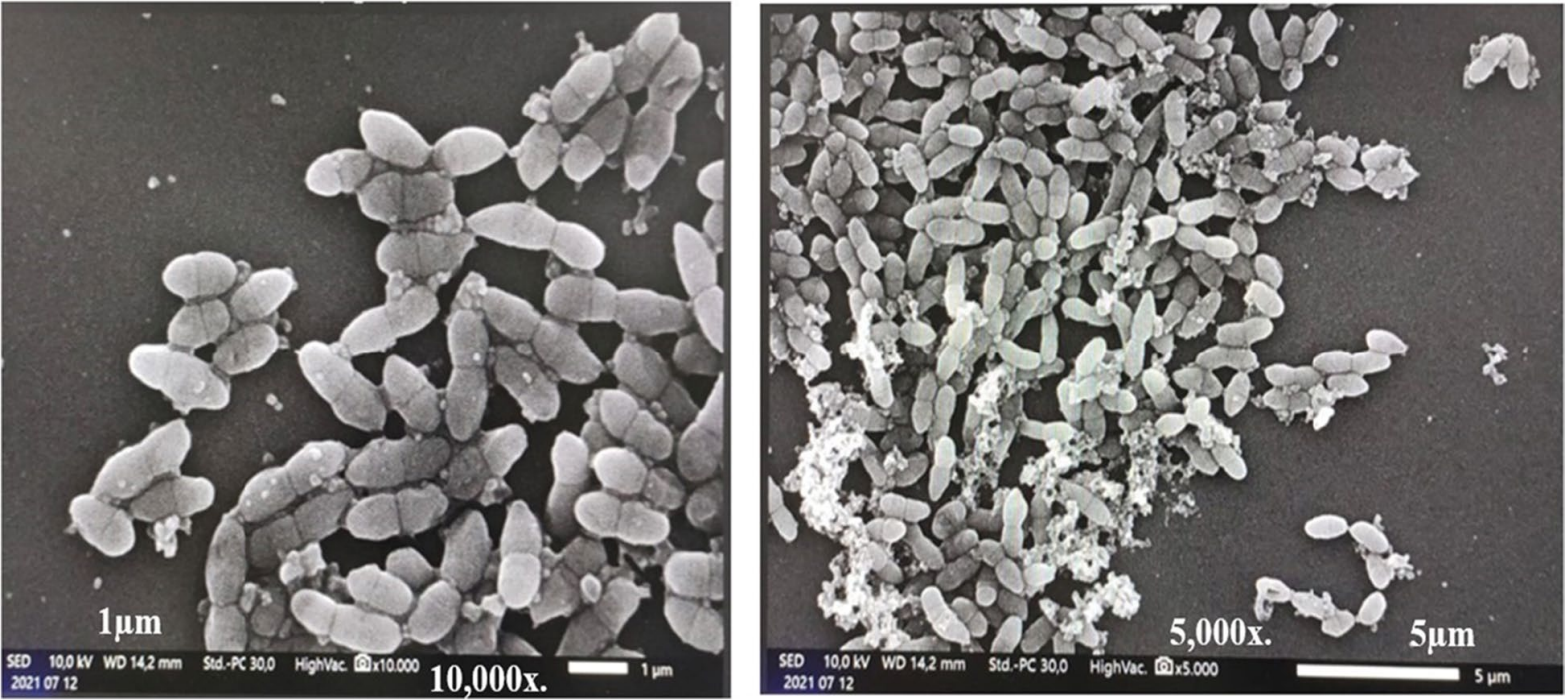
The results of SEM analysis. The length of the cells at a magnification of (× 10,000 is 1 µm and × 5000 is 5 µm)

The Illumina MiSeq Next-generation sequencer was used to produce reads with a total sequence length of 3,801,691 bp ~ (3.8 Mb), with an average GC% content of 37.2%. The expected genome size was approximately ~ 7.6 Mb. The assembled genome of *L. lactis* subsp*. lactis* Lac3 had 55 contigs, with a total length of 2,441,808 bp ~ (2.44 Mb), with an average GC% content of 34.85%, with the largest contig of 639,357 bp, the shortest contig (at N50) of 217,535 bp, and the number of contigs at L50 and L75 were 3 and 6, respectively. There were 2324 genes (2324 CDS) that coded for functional proteins, 56 RNA genes, (5S (2), 16S, 23S) rRNAs, 48 tRNAs, 4 ncRNAs, and 61 (2.5%) pseudogenes out of 2441 total genes. The analysis obtained from the RAST revealed that the genome of *L. lactis* subsp*. lactis* Lac3 contained 238 subsystems. An overview of the subsystem categories for this genome is provided in (Fig. 1). The most represented subsystem features are amino acids and derivatives 77.7% (185), carbohydrates 77.7% (184), protein metabolism 44.11% (105), cofactors, vitamins, prosthetic groups, pigments 44.11% (105), nucleosides and nucleotides 33.1% (79), DNA metabolism 19.74% (47), fatty acids, lipids, and isoprenoids 18.9% (45), cell wall and capsule 15.97% (38), as well as virulence, disease, and defense were 15.9% (38). Besides, phages, transposable elements, and plasmids were 4.6% (11). The identification based on 16S rRNA gene sequencing and the evolution history showed that the strain Lac3 was most closely related to the *Lactococcus lactis* species deposited in NCBI with a sequence similarity ≥ 99.93%. A phylogenetic tree representing taxonomic connections is provided in (Fig. 2). Furthermore, SEM analysis indicated this strain Lac3 of *L. lactis* subsp*. lactis* to have a spherical or ovoid-shaped cell and occurred singly or in chains (Fig. 3).

### Prediction of antibiotic resistance, analysis of pathogenicity, and virulence factors

In the current study, the bacterial whole genome sequence data was used for the prediction of antibiotic resistance and pathogenicity analysis, in which the assembled genome of *Lactococcus lactis* subsp. *lactis* strain Lac3 was uploaded and then analyzed in the CARD and ResFinder online software. The results indicated that neither CARD nor ResFinder detected phenotypic antimicrobial resistance-associated genes encoded in the genome of L. *lactis* subsp. *lactis* strain Lac3. In terms of pathogenicity, *L. lactis subsp. lactis* strain Lac3 was determined to be non-pathogenic, with the matched pathogenic families scoring (0) out of 130 DNA sequences, and its probability of being a human pathogen scoring (0.21 out of 1.0) as predicted using the PathogenFinder web server [24], and no virulence-associated factors were identified using the virulenceFinder tool. Based on the virulenceFinder tool analysis, *L. lactis* subsp. *lactis* Lac3 did not exhibit the well-established virulence determinants or genes coding for an enterococcal surface protein (*esp*), adhesion of collagen (*ace*), serine protease (*sprE*), gelatinase (*gelE*) [21], hemolysin or toxins [25], and cytolysins (*cylL)* [26]. Therefore, the absence of these well-confirmed virulence genes supported the GRAS status of this strain Lac3.

### Mobilome and CRISPR/Cas system prediction

**Fig. 4:**
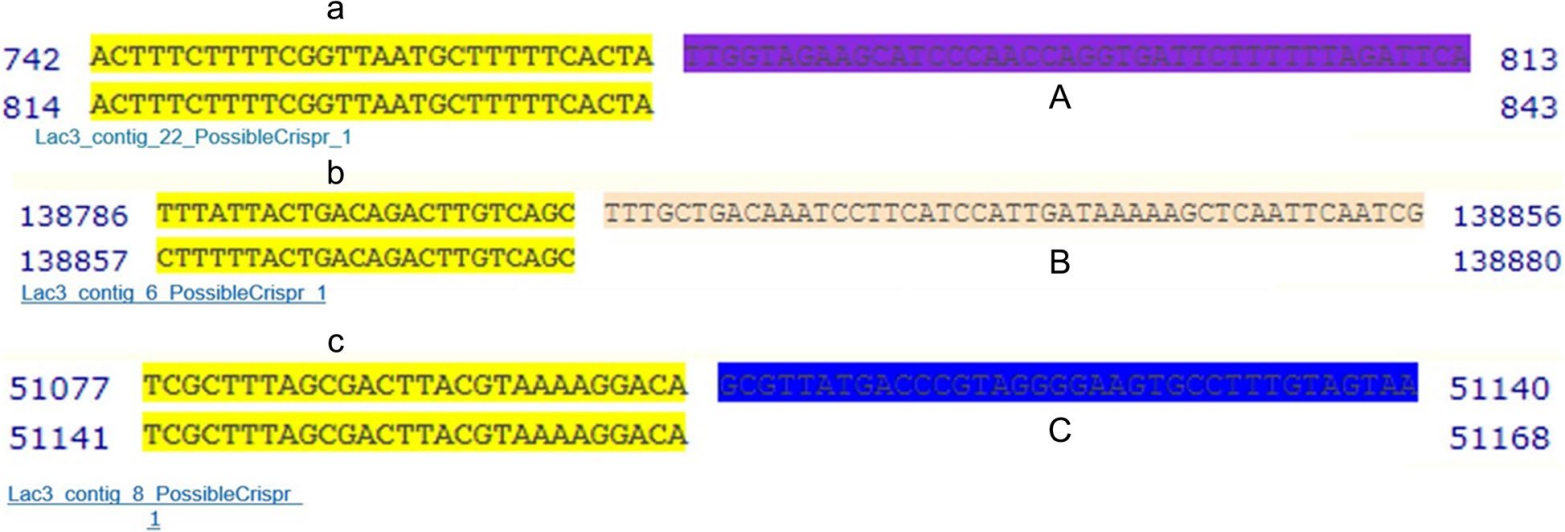
The CRISPR arrays and their spacers were identified in the genome of *L. lactis* subsp*. lactis* Lac3. CRISPR consensus sequences are indicated in yellow with designated letters (**a**, **b**, **c**) and spacer sequences are underlined with designated letters (**A**, **B**, **C**)

This research identified 2 prophage regions (intact and incomplete). One active prophage at contig 2 gene closely related to *Lactococcus* phage 98,202, and one related to *Prochlorococcus* phage MED4-213. Of which, one prophage region was assigned to be intact with a score of (110), whereas other regions were assigned to be incomplete with scores of (30, 30, 20, 20, and 30). The degree of intact and incompleteness was determined as follows: intact (score > 90), questionable (score 70–90), incomplete (score < 70). Several insertion sequences were discovered in the genome of *L. lactis* subsp*. lactis* Lac3 at the threshold of *E*-value greater than one (≥ 1.0) using BLAST algorithm under ISFinder tool in the CGE. The best hits (ISs) producing significant alignment at the threshold, or *E*-value of (0.0), was the IS3 family, included in the group of IS150 and IS6 families. Most encoded proteins (ORF) on these insertion sequences (ISs) are transposases (transposase (TnPA) and putative transposase (InsK) for insertion sequence element IS150). The genome of *L*. *lactis* subsp. *lactis* Lac3 harbored one plasmid designated (repUS33) with a query template length of 1353 bp, located on contig_23 at position (4791…6143). This plasmid shared 99.63% identity and 100% coverage with the plasmid repA (pGdh442) (accession number AY849557). The genome of *L. lactis* subsp. *lactis* Lac3 harbored 3 CRISPR arrays and their respective spacers found on contig_22, contig_8, and contig_6. CRISPR-consensus sequences: 5′-ACTTTCTTTTCGGTTAATGCTTTTTC ACTA-3′; 5′-TTTATTACTGACAGACTTGTCAGC-3′; 5′-TCGCTTTAGCGACTTA CGTAAAAGGACA-3′, respectively. Nonetheless, there were no (CRISPR)-associated Cas proteins detected in these CRISPR arrays (Fig. 4).

### In vitro assessment of probiotic characteristics of *L. lactis *subsp. *lactis* Lac3

#### Antibiotic susceptibility test

In an in vitro study, antibiotic resistance was evaluated using the agar diffusion method (Table 1). The *L*. *lactis* subsp. *lactis* Lac3 was unable to resist all tested antibiotics, except Peniccilin G. This results indicates the absence of acquired antibiotic resistance. The probiotic susceptibility analysis should be the preliminary evaluation when selecting a suitable probiotic candidate.

**Table 1:** The antimicrobial susceptibility test (AST) of *L. lactis* subsp*. lactis* Lac3 using agar diffusion method

*Antibiotic*	*Concentration*	*Inhibition zone diameter (mm)*	*Classification*
Ampicillin	10 µg	22.5 ± 0.41	S
Tetracycline	30 µg	26.2 ± 0.24	S
Amoxicillin	30 µg	22.7 ± 0.24	S
Penicillin G	10 U	15.2 ± 0.24	R
Chloramfenicol	30 µg	22.3 ± 1.89	S

#### Tolerance to NaCl

**Fig. 5:**
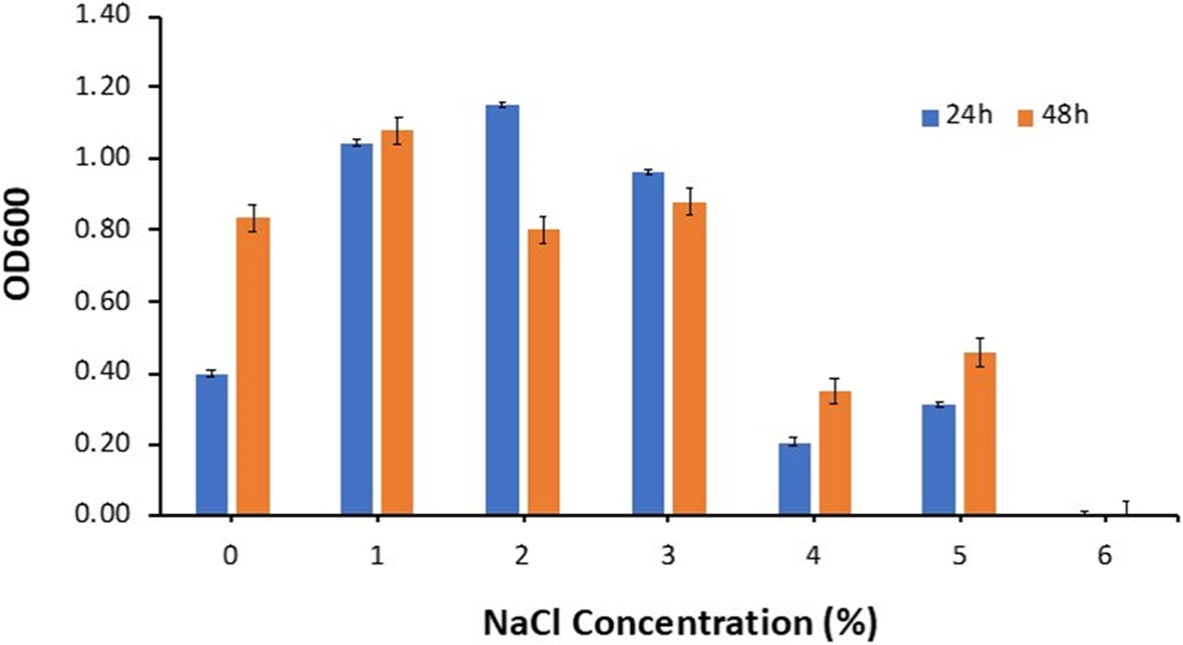
Tolerance of *L*. *lactis* subsp*. lactis* Lac3 to different concentrations of NaCl

*Lactococcus lactis* subsp*. lactis* Lac3 was evaluated for the tolerance against different concentrations of NaCl (Fig. 5). Interestingly, *L. lactis* subsp*. lactis* Lac3 grew normally at concentrations of 1%, 2%, and 3%, but experienced slow growth at concentrations of 4 and 5% NaCl. At 6% NaCl concentration, Lac3 did not grow in any way after both 24 and 48 h of incubation. The current results revealed various proteins associated with osmotic stress responses, including serine protease (DegP/HtrA*)* (EC 3.4.21), ATP-dependent zinc metalloprotease* (*FtsH), glycine betaine, ABC transport system, permease protein* (*OpuAB), and glycine betaine-binding protein (OpuAC).

#### Tolerance to bile salt

Inhibitory effects of bile salt were observed as the bile salt concentration increased, but *L. lactis* subsp*. lactis* Lac3 maintained the growth as the incubation time increased, except after 96 h, when it failed to resist at a bile salt concentration of 2%. Considering the observation of growth and no growth, *L. lactis* subsp*. lactis* Lac3 grew better at bile salt concentrations of (0.3, 0.5, and 1%) (Table 2). A probiotic bacterium living in the gastrointestinal tract of humans must respond to bile salt stress. *L. lactis* subsp*. lactis* Lac3 grew better at bile salt concentrations of (0.3, 0.5, and 1%).

**Table 2:** Tolerance of *L. lactis* subsp*. lactis* Lac3 to different concentrations of bile salts

Growth of strain Lac3 in the presence of bile salt (%)				
Hours	0%(control)	0.3%	0.5%	1.0%
24	+	−	−	−
48	+	+	−	−
72	+	+	+	−
96	+	+	+	+

( −): negative, no growth; ( +): positive, growth; (0%)

#### Tolerance to acid stress

In the current study, the survival of *L. lactis* subsp*. lactis* Lac3 in stressful low pH was observed at (pH 2.5) compared to the control (pH 7.0). The choice of (pH 7.0) as a control was selected to maintain the normal physiological pH in the intestinal tract of humans, which is normally neutral to alkaline [27]. The growth of *L. lactis* subsp*. lactis* Lac3 followed the declining trend as the incubation time increased from 0 to 2 h, meaning that the cell growth declined from (8.39 ± 0.56 Log10 CFU/mL to 7.5 ± 0.3 Log10 CFU/mL) at (pH 2.5). However, the declining trend was considered to be non-significant at (*p* > 0.05) and (*p* = 0.62) (Table 3). Although the growth of *L. lactis* subsp*. lactis* followed a declining trend, this strain Lac3 was not significantly affected by the effects of the acid (*p* > 0.05). Following tolerance against acid, this research predicted L-lactate dehydrogenase (EC 1.1.1.27) and D-lactate dehydrogenase (EC 1.1.1.28), which are responsible for lactic acid production.

**Table 3:** Tolerance of *L. lactis* subsp*. lactis* Lac3 to pH 2.5 and 7.0

pH	Viable cells (Log10 _CFU/_mL)		
	Time (h)		
	0	1	2
2.5	8.39 ± 0.56	8.32 ± 0.56	7.50 ± 0.30
7.0	7.88 ± 0.13	8.07 ± 0.28	7.95 ± 0.10

### Auto-aggregation characteristics

**Fig. 6:**
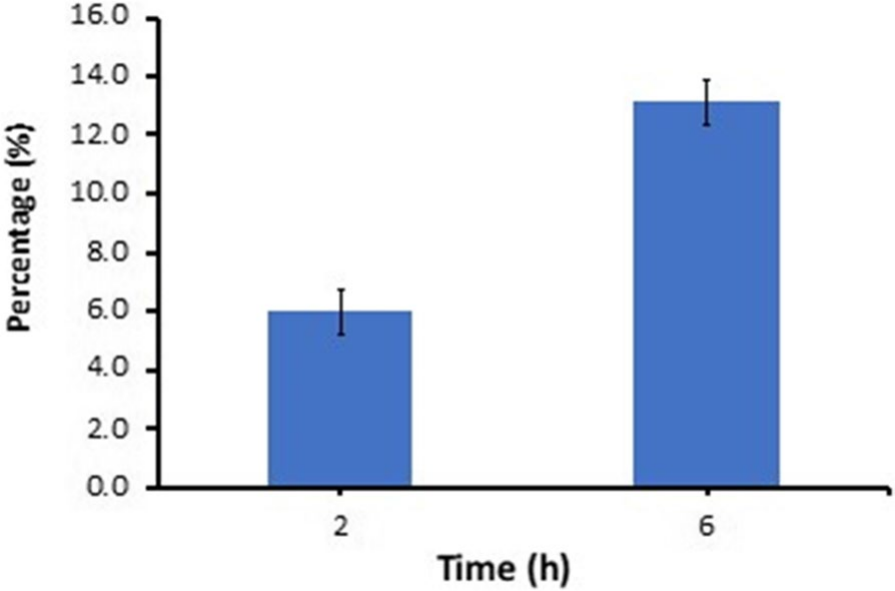
Auto-aggregation ability of *L*. *lactis* subsp. *lactis* Lac3

*Lactococcus lactis* subsp. *lactis* Lac3 demonstrated the ability to adhere to epithelial cells of the intestines and maintain in the human gastrointestinal tract, with an aggregation capacity that increased 6.0 ± 0.76% to 13.1 ± 3.46% from 2 to 16 h of incubation, respectively (Fig. 6).

### Cluster gene analysis

The analysis revealed the predicted cluster gene responsible for the strain's probiotic properties. The results of the analysis can be found in Table 4.

**Table 4:** Predicted cluster gene responsible for probiotic properties from *L. lactis subsp. lactis* strain *Lac3*

Genomic region	Identifier	Protein ID	Product	Length		Function	Location
				NT	AA		
5.1	KB529_07910	MBS3730463.1	S8 family serine peptidase	1293	430	Biosynthetic-additional	35,185-36,477
5.1	KB529_07890	MBS3730451.1	Alpha/beta hydrolase	960	319	Biosynthetic-additional	28,146-29,105
5.1	KB529_07930	MBS3730467.1	Type 2 lantipeptide synthetase LanM	3003	1000	Biosynthetic	39,355-42,357
7.1	KB529_09370	MBS3730748.1	Pyruvate carboxylase	3414	1137	Biosynthetic	20,844-24,257
8.1	KB529_09780	MBS3730822.1	Peptide cleavage/export ABC transporter	2148	715	Biosynthetic	5,495-7,642
8.1	KB529_09790	MBS3730824.1	Lactococcin family bacteriocin	159	52	Biosynthetic	9,209-9,367
21.1	KB529_11715	MBS3731180.1	Lactococcin family bacteriocin	141	46	Biosynthetic	5,407-5,547
28.1	KB529_11960	MBS3731219.1	Lactococcin family bacteriocin	150	49	Biosynthetic	2,736-2,885

## Discussion

The *Lactococcus lactis* subsp. *lactis* strain Lac3 bacterial whole genome was sequenced, assembled, annotated, and deposited in the NCBI database. Genome sequencing and omic-based approaches are robust strategies to predict potential probiotic associated-functional genes involved in genome plasticity and stability, metabolic pathways, identification of antimicrobial resistance phenotypes, virulence factors, and safety. Furthermore, in vitro, in vivo, and omic studies have been used to determine the suitable probiotic microorganisms and their associated probiotic potentials, including tolerance to acidic conditions, bile salts, antimicrobial compounds, immuno-modulation and adhesion mechanisms, etc. [28, 29]. Probiotics provide health benefits to their hosts by modulating the intestinal microbiota and thus inhibiting pathogen colonization; modulating the host immunity response; lowering serum cholesterol levels; exerting antihypertensive, antidiabetic, and antioxidant effects; and producing bacteriocins [7].

In the current study, the genome analysis was performed for the prediction of antimicrobial resistance, pathogenicity, the CRISPR/Cas system, virulence determinants, and mobile genetic elements, including plasmids and insertion sequences (IS). Phylogenetic and genome analysis and scanning electron microscopy were used for the strain identification. The results showed that the strain designated Lac 3 belongs to the *Lactococcus lactis* species. The concern about antibiotic resistance should arise because the microbiota might acquire and transfer antibiotic resistance genes to pathogenic bacteria interchangeably [30]. Based on genome analysis using CARD and ResFinder-online bioinformatics tools, the result revealed the absence of antibiotic resistance-associated genotypes and phenotypes. In addition, the in vitro study was performed using the antibiotic susceptibility test, which showed that the strain Lac3 was highly susceptible to all 4 tested antibiotics but slightly resistance at the lowest threshold to Penicillin G (Table 1). In accordance with previous report [19], antibiotic susceptibility to ampicillin, chloramphphenicol, erythromycin, gentamicin, penicillin, and vancomycin is a common intrinsic characteristic of *L. lactis*. However, other research has revealed that some *L. lactis* strains are penicillin G resistant. This result probably correlated with the impact of the environment or stress that experienced by the bacteria [31]. Concerning pathogenicity, the current results were consistent with the safety status and agreed with other studies. For instance, five *Lactobacillus plantarum* strains (currently renamed to *Lactiplantibacillus plantarum*) isolated from a fermented chicha were reported to be non-pathogens at a probability of 0.08 out of 1 [8]. Also, *Lactobacillus reuteri* (recently renamed to *Limosilactobacillus reuteri*) PNW1 was predicted as a non-human pathogen at a probability of 0.217 and matched pathogenic families of zero (0) [32].

The CRISPR/Cas system confers immunity or a barrier against the foreign DNA invaders in bacteria [33], and the presence of the CRISPR/Cas system and its associated spacers could be an advantage for the newly identified strain Lac3 for its protection. The analyses of mobilome provide insight into genome stability, adaptability, and evolution or whether the host probiotics are likely to acquire and transfer new genes, including antibiotic resistance genes. However, if the genome of a probiotic harbors mobile genetic elements (MGEs) and associated antibiotic resistance genes, it should not be used as a probiotic strain due to the possibility of transferring resistance genes through conjugation or other mechanisms [34]. Interestingly, the predicted mobile genetic elements were not associated with antibiotic resistance or harmful factors as predicted by MGEFinder. Our study revealed that most identified prophages were functional phages that harbor attachment sites *attL* and *attR*. Dislike, the absence of the attachment sites *attL* and *attR* in a prophage implies defective and non-functional phages [35]. However, prophages contribute to the genome plasticity of the host where they increase the metabolism and physiology of the host by modulating the growth and survival of the host in the gastrointestinal environment, as well as protecting the host cell from virulent invader bacteriophages and antimicrobial compounds [33]. From the assumption of EFSA guidelines, a probiotic that does not harbor the transferable antibiotic resistance genes and does not carry the virulence factors like *IS16*, enterococcal surface protein (*esp)*, and (*hyl*) (hyaluronidase) should be recommended as a feed additive. Nevertheless, the presence of well-confirmed virulence factors excludes a probiotic from exploitation [36]. Importantly, *L. lactis* subsp. *lactis* Lac3 did not exhibit well-identified virulent determinants (genes) encoding for enterococcal surface protein (*esp*), adhesion of collagen (*ace*), serine protease (*sprE*), gelatinase (*gelE*) [21], hemolysin or toxins [25], and cytolysins (*cylL)* [26]. This safety analysis was confirmed by using (Virulence-Finder 2.0) to the threshold of 90%, a minimum length of 60%, and using reference organisms: *Listeria*, *Staphylococcus aureus,* and *Escherichia coli* as default parameters, assessed from the (CGE) (https://cge.cbs.dtu.dk/services/VirulenceFinder/) [26]. Therefore, the absence of these well-confirmed virulence genes supported the GRAS status of the *L*. *lactis* subsp. *lactis* strain Lac3.

In the current study, we conducted in vitro assessment of probiotic characteristics of *L. lactis* subsp*. lactis* strain Lac3. The Lac3 strain showed normal growth at 1–3% NaCl concentration and start to grew slowly at 4–5% concentration. Our results are consistent with that of Gonzalez Duran [37], who revealed the normal growth of *Lactococcus lactis* R-604 up to 5% concentration of NaCl. However, *L*. *lactis*, NTH4 exhibited a better growth at the concentration of NaCl of 10%. Therefore, these discrepancies are under the concept that salt tolerance is strain-associated [38].

Bile salt is considered as the parameter to evaluate for selecting a suitable probiotic because bile salt compounds can inhibit the metabolism of microbiota living in the gastrointestinal tract of humans by disrupting the bacteria membrane and exerting oxidative stress, which causes DNA damage and protein denaturation [39]. The obtained results agreed with the normal physiological concentration of bile salt in the intestines (0.05–2%) [40]. Our findings were also in line with Jatmiko et al. [41], in which sixteen strains of *L. lactis subsp. lactis* were adapted to grow better at bile salt (Oxgall) concentrations of (0.5%, 0.3%, 1%)*.*

For the probiotics to live in the intestines of humans, they must be able to tolerate the acidic condition of the stomach during the gastrointestinal transition, which has been reported to vary between 2.5 and 3.5, except in the case of prolonged fasting or after taking a meal, where it can rise as high as 4.5 [19]. This assumption confirmed the probiotic property of the strain Lac3 because of its ability to grow at low (pH 2.5). Following the acidity tolerance characteristic, this research predicted L-lactate dehydrogenase (EC 1.1.1.27) and D-lactate dehydrogenase (EC 1.1.1.28), which play a great role in maintaining NAD + /NADH and increasing ATP production, resulting in the proton extrusion and formation of ATPase, therefore helping bacteria tolerate acid [42].

Aggregation and auto-aggregation are desirable properties necessary for the probiotics to adhere to the area of colonization, including the gastrointestinal tract humans [7]. The adhesion ability to the epithelial surface indicates the capability of a probiotic to exert exclusion competition against pathogens and immunomodulation mechanism. Similar to Abushelaibi et al. [43], *L. lactis* subsp*. lactis* strain Lac3 showed the adhesion ability ranging between 0.1–10% and 0.6–38%. The fact that the strain Lac3 has probiotic potential, lacks antibiotic resistance-associated genes and virulence factors, and does not belong to the pathogen family suggests that it has medical and nutritional applications. However, genomic and in vitro analyses alone are insufficient to confirm its health benefits; thus, additional in vivo experiments are required to reveal the specific health benefits. One of the common properties of probiotic bacteria is their ability to generate natural antibiotic-like substances called bacteriocin. Bacteriocin has been reported to play a role in the antimicrobial activity of the lactic acid bacteria (LAB), together with organic acids and hydrogen peroxide [44–46]. Type 2 lanthipeptide synthetase LanM is another protein that has antimicrobial properties in the Lac3 strain. Lanthipeptides that display antimicrobial activity are called lantibiotics [47]. Aside from antimicrobial activity, we also look into other probiotic properties related to bacterial-environment interaction, such as adhesion capability, auto- and co-aggregation, and safety properties (antibiotic resistance and the absence or presence of virulence factor). The bacteriocin family is well-known for its antimicrobial properties. Meanwhile, the serine peptidase family and alpha/beta hydroxylase family are associated with the interaction between LAB and the environment, for example, with adherence to host epithelial cells and auto-aggregation mechanisms [46, 48, 49].

## Conclusion

The potential probiotic properties of *L. lactis* subsp*. lactis* strain Lac3 were associated with its ability to survive in stressful conditions, including acid, bile salts, NaCl, and the ability to form auto-aggregation. In the current study, there were no virulence factors and pathogenicity associated with *L. lactis* subsp*. lactis* Lac3, which highlights its beneficial properties to be exploited as a probiotic candidate. The presence of mobile genetic elements contributes to the genome plasticity and evolution of this strain Lac3. *L*. *lactis* subsp. *lactis* strain Lac3 was susceptible to all tested antibiotics, which reveals that it does not carry antibiotic resistance genes.

## Data Availability

The complete DNA sequence and the corresponding annotation of *L. lactis* subsp*. lactis* Lac3 is available in the NCBI GenBank (accession number JAGRPZ000000000). The Plasmid sequence and 16S rRNA gene sequence are available on (accession number JAGRPZ010000023.1 and MZ618669), respectively in the NCBI. The raw Sequencing Read Archives (SRA) was deposited in the NCBI (accession number SRR14690701). This whole-genome shotgun project was deposited at DDBJ/ENA/GenBank under (accession number JAGRPZ000000000). The version described in this paper is version (JAGRPZ000000000).

## Abbreviations

LAB: Lactic acid bacteria
GRAS: Generally recognized as safe
FDA: The United States Food and Drug Administration
EFSA: The European Food Safety Authority
WGS: Whole-genome sequencing
LIPI: Indonesian Institute of Sciences
rRNA: Ribosomal RNA
BLAST: Basic local alignment search tool
NCBI: National center for biotechnology information
SEM: Scanning electron microscopy
PATRIC: Pathosystems resource integration center
PGAP: Prokaryotic genome annotation pipeline
RAST: Rapid annotation using subsystem technology
CARD: Comprehensive antibiotic resistance database
MGEs: Mobile genetic elements
IS: Insertions sequences
CGE: Center for Genomic Epidemiology
CRISPR: Clustered regularly interspaced short palindromic repeats
AST: Antibiotic susceptibility test
MRS: De Man and Rogosa Sharpe
TPC: Total plate count
SRA: Sequencing read archives
ORF: Open reading frame
AMR: Antimicrobial resistance
